# 3DOF Ultrasonic Motor with Two Piezoelectric Rings

**DOI:** 10.3390/s20030834

**Published:** 2020-02-04

**Authors:** Vytautas Jūrėnas, Gražvydas Kazokaitis, Dalius Mažeika

**Affiliations:** 1 Faculty of Mechanical Engineering and Design, Kaunas University of Technology, Studentų str. 56, 51424 Kaunas, Lithuania; vytautas.jurenas@ktu.lt; 2 Faculty of Fundamental Sciences, Vilnius Gediminas Technical University, Saulėtekio av. 11, 10221 Vilnius, Lithuania; dalius.mazeika@vgtu.lt

**Keywords:** piezoelectric multi-DOF actuator, ultrasonic motor, spherical rotor, electrode segmentation, finite element analysis, ring-shaped piezoelectric transducer

## Abstract

A novel design of a multiple degrees of freedom (multi-DOF) piezoelectric ultrasonic motor (USM) is presented in the paper. The main idea of the motor design is to combine the magnetic sphere type rotor and two oppositely placed ring-shaped piezoelectric actuators into one mechanism. Such a structure increases impact force and allows rotation of the sphere with higher torque. The main purpose of USM development was to design a motor for attitude control systems used in small satellites. A permanent magnetic sphere with a magnetic dipole is used for orientation and positioning when the sphere is rotated to the desired position and the magnetic field synchronizes with the Earth’s magnetic dipole. Also, the proposed motor can be installed and used for robotic systems, laser beam manipulation, etc. The system has a minimal number of components, small weight, and high reliability. Numerical simulation and experimental studies were used to verify the operating principles of the USM. Numerical simulation of a piezoelectric actuator was used to perform modal frequency and harmonic response analysis. Experimental studies were performed to measure both mechanical and electrical characteristics of the piezoelectric motor.

## 1. Introduction

Piezoelectric actuators and devices are widely used for various applications. It is possible to find them in photo cameras, laser beam control mechanisms, optical systems, robotics, etc. [1]. One of the possible areas, where the piezoelectric actuator could be used for specific tasks is the space industry. Piezoelectric actuators are small and reliable and can be adapted for specific necessary applications. Requirements for equipment that will be used for space applications are very strict—it includes space, mass and volume, complexity and many other parameters for the assets, which will be used for the missions. Piezoelectric actuators are superior for such kind of applications as high precision positioning systems for the adjustment and control of optical systems, telescopes and other fine instruments [2]. Piezoelectric actuators have a high power/volume ratio, generating high force or torque and also being sufficiently small. The mass of the actuators is fine, and they have low inertia and fast response times. Also, piezoelectric actuators are non-magnetic and can operate in strong magnetic fields or can be used when the system should produce as little as possible magnetic noise. Also, they have high repeatability and ability to work in a micrometer or even nanometer scale, including high frequencies, that allows them to integrate and use piezoelectric actuators in a wide variety of tasks. The benefits of piezoelectric actuators allow changing typical drives or complex actuators that are used for specific applications and simplify the whole system design.

Typically, piezoelectric actuators have only one degree of freedom (DOF), but actuators also exists with several DOF [3]. Drives with multi-DOF actuators operate by the accumulation of small steps into the unlimited rotation of the rotor around a different axis or translation motion of the slider in different directions.

There exist several multi-DOF USMs developed to orientate and position the object in several directions. A 3-DOF Langevin type piezoelectric actuator was developed by Zhao et al. It consists of a cylindrical stator and spherical rotor. This actuator operates when employs the superposition of bending and longitudinal vibration modes, when the harmonic signal is applied to the three groups of piezoelectric rings [4]. Piezoelectric actuator with three DOF rotation of the sphere was created by Vasiljev et al. [5]. Such actuators consist of the cylindrical or ring-type stator and spherical rotor. The operation of the actuator is based on the shaking beam principle. The design of the actuator is based on a vibrating beam-type frame, several piezoceramic stacks, and overlays. Four driving tips are mounted on the frame and are in contact with the rotor. A particular deformation mode of the actuator is excited when the harmonic electric signal of the resonant frequency is applied [6]. Shi et al. [7] presented a MDOF humanoid eyeball USM orientation system which consists of a piezoelectric actuator and a spherical rotor. This system is based on various deformation modes of the piezoelectric ring-type actuator and spherical rotor which relies on four supports.

The novel design concept for three DOF piezoelectric ultrasonic motor of rotational motion is proposed and analyzed in this paper. Two ring type piezoelectric actuators are used for the rotation of the spherical rotor. Numerical simulations and experimental measurements were executed to verify principles and considerations of output characteristics of the developed actuator. The results of numerical and experimental studies are presented.

## 2. Design Concept and Operating Principle

The novel design of piezoelectric actuator USM includes two ring-type piezoelectric transducers, a spherical rotor, and holder. The spherical rotor is inserted between two piezoelectric ring-type transducers and is driven by employing the radial vibrations of the transducers. Such a piezo-mechanical system has the advantage of increasing the mechanical force which spins the spherical rotor. The implementation of this setup can be used for various applications where precise displacement is needed, i.e., space applications, nano-satellites, laser beam control systems, and robotics [8].

**Figure 1:**
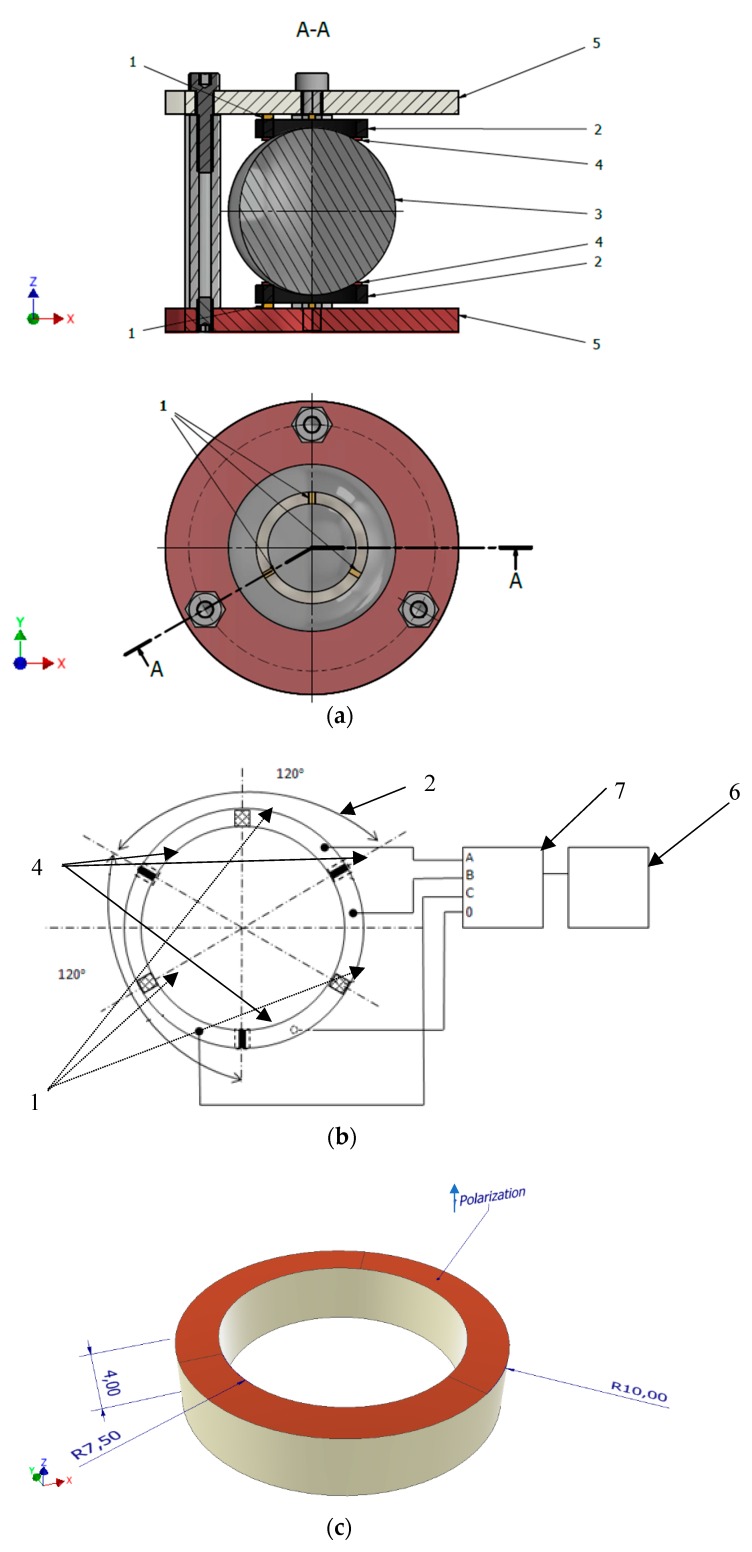
(**a**) 3DOF USM with the spherical rotor and two transducers: schematic view; (**b**) electrical layout and arrangement of electrodes of the ring type transducer; (**c**) Detailed view of piezoelectric transducer.

The principle design of the proposed USM is shown in Figure 1a. The spherical rotor (3) is placed between two ring type piezoelectric transducers (2). Both transducers are identical and are made from “hard-type” PZT-4 type piezoelectric piezoceramics [9,10]. Transducers are polarized along with the thickness i.e., in the Z-axis direction. Electrodes are located on the top and bottom surface of the transducer. The top surface electrode is divided into three equal sections arranged at every 120° while the electrode located at the bottom surface is not segmented and is used for grounding. Three friction-resistant contact elements (4) are glued at the top surface of the transducer and are arranged in such a way that the angle between them is 120°. These contact elements are used to drive the rotor. The three spacers (1) are glued between ring type transducers (2) and flanges (5) and are used for uniform preload of the friction-resistant elements (4). Also, they operate as vibration dumpers between the transducer (2) and flange (5). The flanges are joined together by the three bolts with certain preload force.

The electrical setup of the drive is shown in Figure 1b and includes a signal generator (6) and the control unit (7). The control unit is connected with the three pairs of electrodes. Every pair of electrodes are connected to the channels A, B, and C, respectively. Only one channel is active at a given time. This setup allows excite one segment of piezoelectric actuators and induce an elliptical displacement motion of the contact element (4).

The operation of USM is based on the excitation of the radial vibration mode of both piezoelectric rings. The USM generates a 3DOF rotary motion of the spherical rotor when the harmonic electric signal of the particular resonance frequency is applied to piezoelectric transducer electrodes. An elliptical motion of the contact points between rotor and ring type transducers is achieved and the rotor receives a rotary force from the stator through friction. The motion characteristics of the rotor, including velocity and step size, are controlled by means of electric signal amplitude and signal duration. Rotation direction is changing by switching the driven electrodes of the top and bottom piezoelectric ring type transducers.

## 3. Modeling of the Actuator

Numerical modeling was performed to find the operating resonant frequency and vibration mode of the actuator. The segmentation and size of electrodes of the piezoelectric transducer were analyzed as well to investigate the dependence of the contact point motion trajectory when the transducer has a different configuration of electrodes.

**Figure 2:**
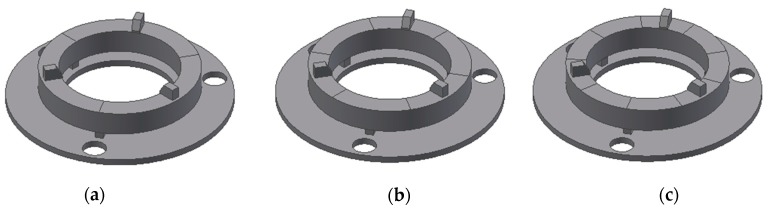
3D models of the ring-shaped transducer with segmented electrodes: (**a**) 3 sectors of 120°; (**b**) 6 sectors of 60°; (**c**) 9 sectors of 40°.

The simulation of the ring type actuator was divided into two steps. First of all, a modal analysis of the actuator was performed. Based on the results of modal analysis, harmonic response analysis was made. This analysis was performed using three different configurations of electrodes and the obtained results were compared. Top surface electrodes were divided into three, six, or nine equal sectors as shown in Figure 2.

### The Actuator

Actuator of the motor contains piezoelectric ring type transducer, contact elements, and flange. A 3D geometrical model of the piezoelectric actuator was created using Autodesk Inventor 2018 and COMSOL Multiphysics 5.4 was used to run simulations. 3D model contains piezoelectric transducer, contact elements and flange. Sphere and upper side components of USM were not included into simulations. Dimensions of the piezoelectric transducer are as follows: outer diameter is 20 mm, inner diameter is 15 mm and height is 4 mm. The transducer is made from PZT-4 piezoelectric ceramic. This type of ceramic has a perovskite polycrystalline structure and each crystal consists of a small tetravalent metal ion in a lattice of large divalent metal ions. Usually, the small tetravalent ion is a titanium or zirconium. The large divalent metal ion is a lead [11,12,13]. Material properties of the transducer are shown in Table 1.

The flange has a outside diameter of 30 mm, diameter of center hole is 14 mm and thickness is 0.8 mm. Size of the contact elements are as follows: length is 2.2 mm, width is 1 mm, and height is 1.5 mm. The material of flange is aluminum and material of the contact elements was aluminum oxide (Al_2_O_3_). Material properties are given in Table 2 [14,15].

The finite-element method was used to perform modal frequency and harmonic response analysis, to calculate trajectories of the contact elements. Basic dynamic equations of the piezoelectric actuator are derived from the principle of minimum potential energy by means of variational functionals and can be written as follows [16]:

(1) $$\{\begin{array}{c} M\ddot{u}+C\dot{u}+Ku+T\varphi =F,\\ T^{T}u+S\varphi =Q, \end{array}$$

where *M*, *K*, *T*, *S*, and *C* are matrices of mass, stiffness, electroelasticity, capacity and damping, respectively; *u*, *φ*, *F*, and *Q* are, respectively, the vector of nodes displacements, potentials, external mechanical forces, and charges coupled on the electrodes. The driving force of the actuator is obtained from the piezoelectric transducer. The finite element discreetisation of the transducer consists of elements with the nodes coupled with electrodes that have the known values of electric potential. The nodal electric potential of remaining elements is calculated during solution. The dynamic equation of the piezoelectric actuator in this case can be expressed as follows:

(2) $$\{\begin{array}{c} M\ddot{u}+C\dot{u}+Ku+T_{1}\varphi_{1}+T_{2}\varphi_{2}=F,\\ T_{1}^{T}u-S_{11}\varphi_{1}-S_{12}\varphi_{2}=Q_{1},\\ T_{2}^{T}u-S^{T}{}_{12}\varphi_{1}-S_{22}\varphi_{2}=0, \end{array}$$

where:

$$T=\left[T_{1}\;T_{2}\right],$$

$$S=\left[\begin{array}{cc} S_{11} & S_{12}\\ S^{T}{}_{11} & S_{22} \end{array}\right]$$

where *φ*_1_, *φ*_2_ are the vectors of nodal potentials of nodes coupled with electrodes and the vector of nodal potentials calculated during numerical simulation, respectively. Natural frequencies *ω* and modal shapes of the actuator are derived from the modal solution of the piezoelectric system:

(3) $$\det(K^{*}-\omega^{2}M)=0$$

where K* is a modified stiffness matrix. In case when *Q*_1_ = 0 it can be written as follows:

(4) $$K^{*}=K+T\;S^{-1}T^{T}$$

When *φ*_1_ = 0 the modified stiffness matrix is:

(5) $$K^{*}=K+T_{2}S_{22}^{-1}T_{2}^{T}$$

Harmonic response analysis of the piezoelectric actuator is carried out applying sinusoidal varying voltage with different phases on electrodes. Due to the inverse piezoelectric effect, corresponding mechanical forces are obtained:

(6) $$F_{1}=\left(T_{2}\;S_{22}^{-1}S_{12}^{T}-T_{1}\right)U\sin\left(\omega_{k}t\right)$$

where *U* is a vector of voltage amplitudes, applied to the nodes coupled with electrodes.

Modal analysis was performed when the piezoelectric actuator was clamped while short circuit boundary conditions were applied for the electrodes. During harmonic response analysis, the electric signal was applied for one segment of the piezoelectric actuator and the system was constrained using three external holes. Further, 5 N force was applied which mimics the load of the rotor and preload of the connecting bolts. Piezoelectric and dielectric losses were neglected in the model. Displacement trajectories were analyzed of the points located on the top surface of the contact elements.

**Figure 3:**
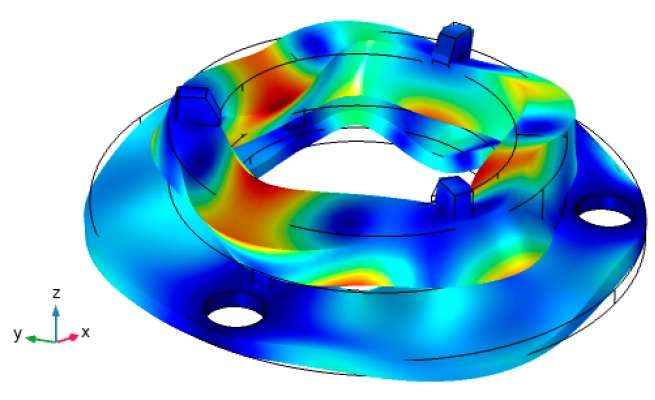
The modal shape of the actuator at 91.15 kHz.

Modal analysis was carried out to find the natural frequencies and modal shapes. Determination of deformations of the actuator in X, Y, and Z directions allow finding resonant frequencies and 3D vibration modes for the rotation of the sphere in all directions. Analysis of the oscillations in a wide frequency range identified several resonance modes when the rotation of the sphere is expected and dominant displacements of the transducer are in radial and axial directions. These types of vibrations generate an elliptical displacement motion of contact points of the ring type transducer and the rotational motion of the sphere is achieved. While analyzing the oscillation modes of the actuator, it was found that the piezoelectric actuator has a mode that is appropriate for the rotation of the sphere. The modal shape at the frequency of 91.15 kHz is shown in Figure 3.

**Figure 4:**
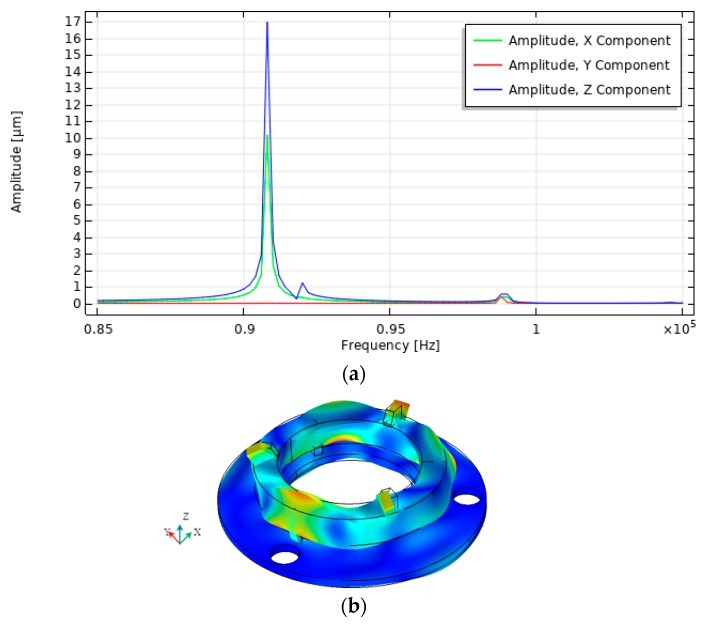
Results of harmonic response analysis when the top electrode is divided into three equal segments; (**a**) amplitude-frequency characteristic; (**b**) vibration modes at 90.80 kHz.

**Figure 5:**
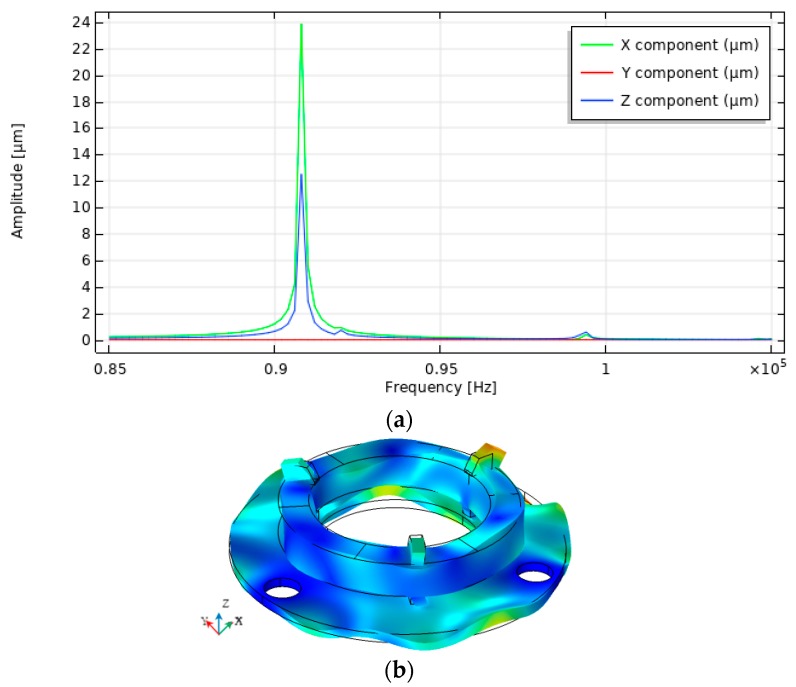
Results of harmonic response analysis when the top electrode is divided into six equal segments: (**a**) amplitude-frequency characteristic; (**b**) vibration modes at 90.80 kHz.

**Figure 6:**
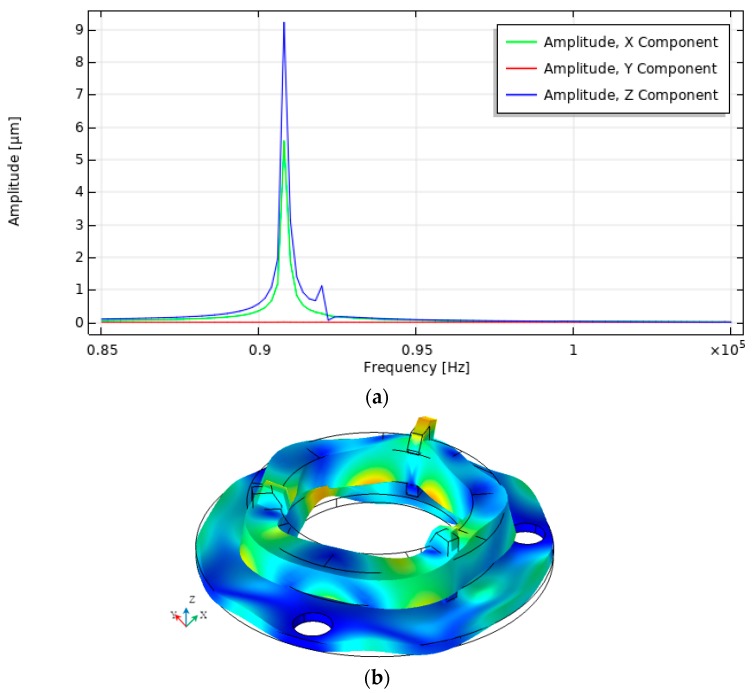
Results of harmonic response analysis when the top electrode is divided into nine equal segments. (**a**) amplitude-frequency characteristic; (**b**) vibration modes at 90.80 kHz.

A harmonic response analysis was performed to determine the response of the contact element motion when a voltage of 100 V was applied to one of the segmented electrodes. The simulation was done at the frequency range from 85 kHz to 105 kHz with the solution steps at 50 Hz. Calculations were performed when only one electrode section was excited because the system is symmetrical and at a certain time only one contact element is used to rotate the sphere. Three scenarios were investigated, i.e., when the top electrode is divided into three, six or nine equal sections. The influence of the electrodes configuration on the vibration amplitudes in X, Y, and Z directions was analyzed. Calculated contact point vibration amplitudes versus frequency are given in Figure 4, Figure 5 and Figure 6.

**Figure 7:**
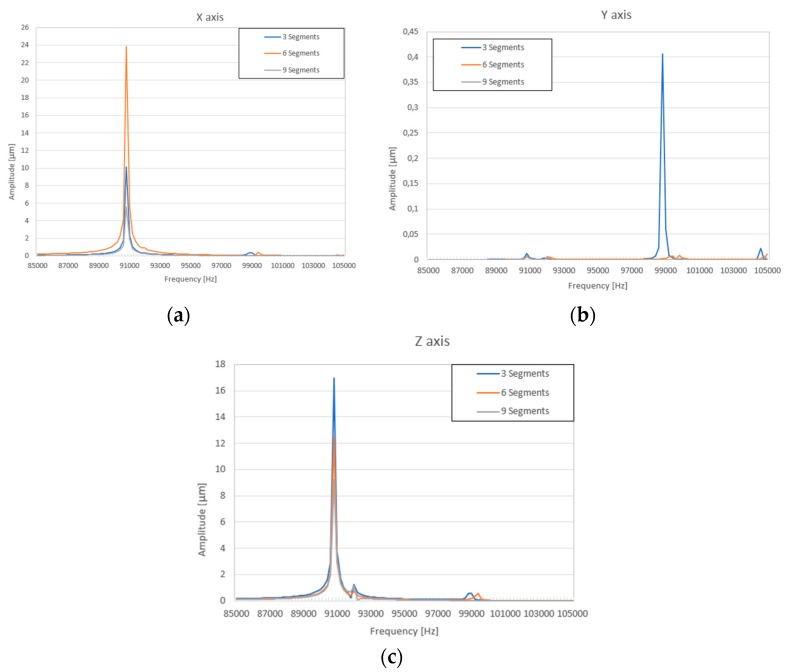
Summarized results of amplitude-frequency characteristic with different segmentation of electrode: (**a**) displacement amplitude in x direction; (**b**) displacement amplitude in y direction; (**c**) displacement amplitude in z direction.

Summarized results of amplitude-frequency characteristic when three, six, and nine segments are used, as presented in Figure 7.

The graphs show that the vibration amplitudes are dominating in *X* and Z directions and the number of segmented electrodes influences the amplitudes of vibration. The highest oscillation amplitude in X direction equal to 23.9 µm and is achieved when the top electrode is divided into six segments. It is 2.4 times larger value compared to the amplitude obtained when the electrode has three segments. The highest amplitude in Z direction equal to 17.1 µm is obtained when the top electrode is divided into three segments and it is 25% bigger compared to the amplitude when the electrode is divided into six sections. Also, it must be noticed that vibration amplitude in the Y direction is close to zero at the frequency of 90.80 kHz, but the amplitude peak can be seen at the frequency of 99.21 kHz, Figure 7b. The amplitude value at this peak is 0.41 µm and it is much smaller compared to the amplitudes of vibration in X and Z directions shown in Figure 7a,b at the frequency of 90.80 kHz. Also, this peak indicates different vibration mode than is shown in Figure 4, Figure 5 and Figure 6.

**Figure 8:**
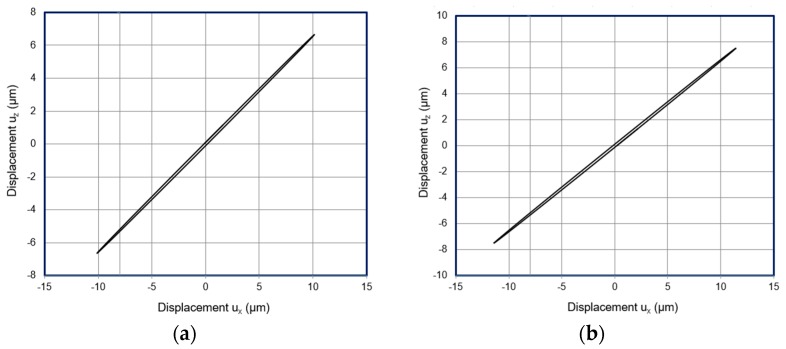

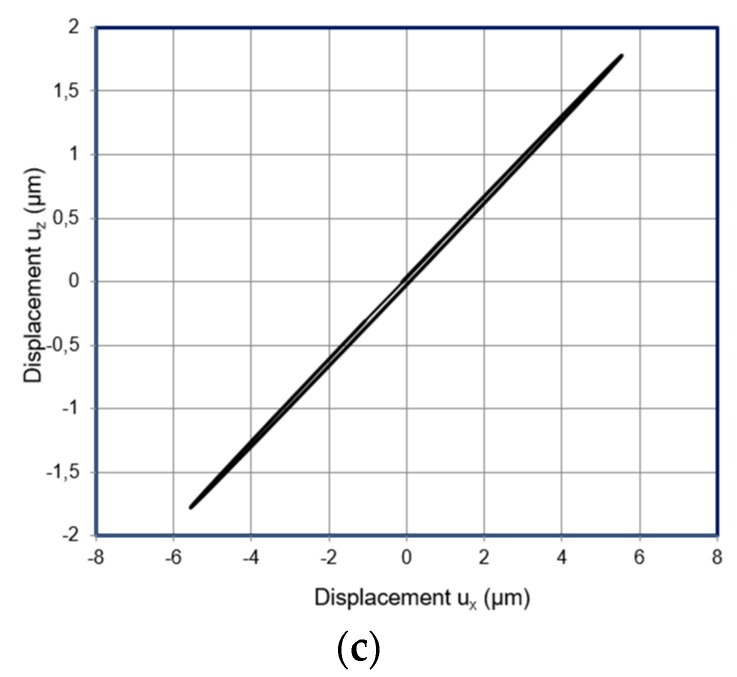
Contact point vibration trajectories of the ring type actuator. (**a**) 3 electrodes; (**b**) 6 electrodes; (**c**) 9 electrodes.

Trajectories of the contact point were calculated as well and presented in Figure 8. It can be noticed that the trajectories of the contact point are elliptical shaped. The length of the major and minor axes are presented in Table 3. It can be seen that largest length of the axes is obtained when 6 segments are used. Numerical simulations of the piezoelectric USM actuator confirmed that elliptical trajectories of the contact point can be achieved and actuator can be used for rotation of the sphere.

## 4. Experimental Study

**Figure 9:**
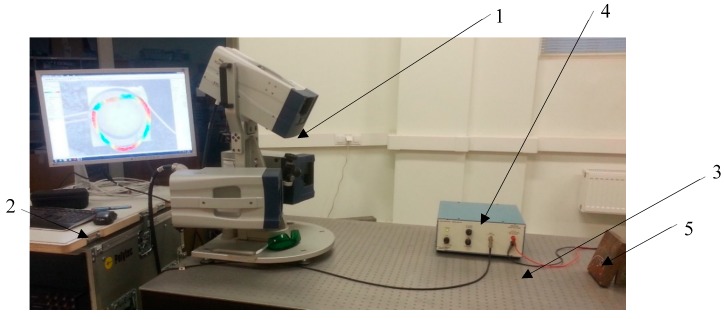
Experimental setup with Polytec Laser Doppler Scanner. 1—PSV-500 3D scanning Head of the vibrometer; 2—PSV-500 3D scanning vibrometer and PC; 3—optical table; 4—amplifier EPA-100 (PiezoSystems Inc., Hopedale, MA, USA); 5—Steel plate with the mounted piezoelectric ring. The piezoelectric ring is mounted on a metal plate and fixed with double-sided adhesive tape.

**Figure 10:**
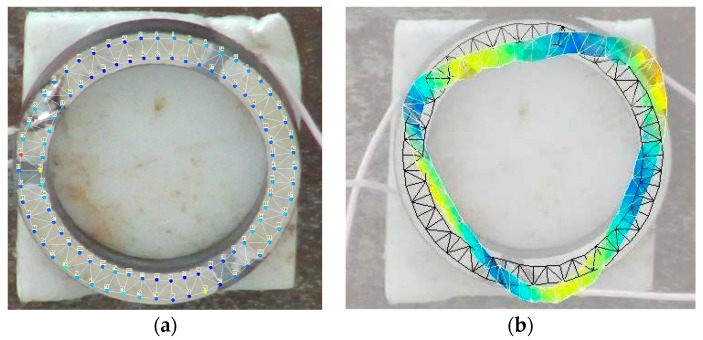
Prototype transducer (**a**) and measuredvibration shape of the transducer at the frequency of 91.6 kHz; (**b**) measured shape of the transducer vibrations.

A prototype of 3-DOF actuator was made according to the dimensions used in the numerical model to validate the feasibility and operating principle of the USM. The material properties that were used for this prototype are presented in Table 1. Experimental measurements were performed using Polytec PSV-500-3D-HV 3D scanning laser vibrometer, signal amplifier EPA-104 and impedance analyzer 6500B [17,18,19]. The setup is shown in Figure 9, while a photo of the transducer is shown in Figure 10a. The input voltage of the frequency range from 0.01 to 150 kHz was applied to one of the electrodes.

**Figure 11:**
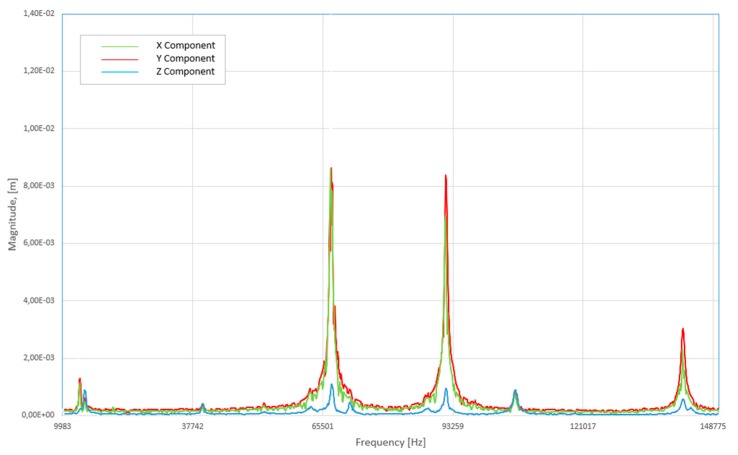
Versus frequency graph with the peak oscillation values at 65 and 91.6 kHz: red—X coordinate, green—Y coordinate, blue—Z coordinate.

Firstly, the area of the flat surface was scanned and the mesh of this surface was created. Further steps were scanning and measuring deformation characteristics of the prototype during frequency variations and the creation of 3D model. These measurements allow defining amplitudes of vibrations. The measured shape of the transducer vibrations can be seen in Figure 10b. The measurement results are shown at the frequency of 91.6 kHz. The measured vibration amplitudes are shown in Figure 11. It can be noticed that the shape of the measured oscillation mode is similar to that achieved during numerical simulations.

**Figure 12:**
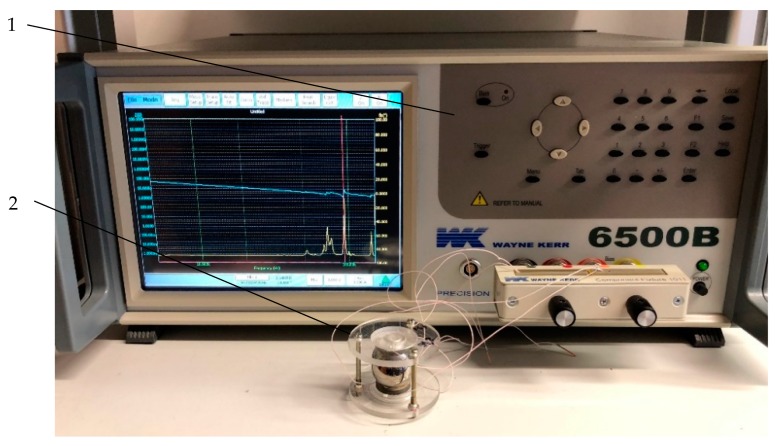
The setup for impedance analysis. 1—Kayne Kerr 6500B impedance analyzer; 2—USM with spherical rotor.

**Figure 13:**
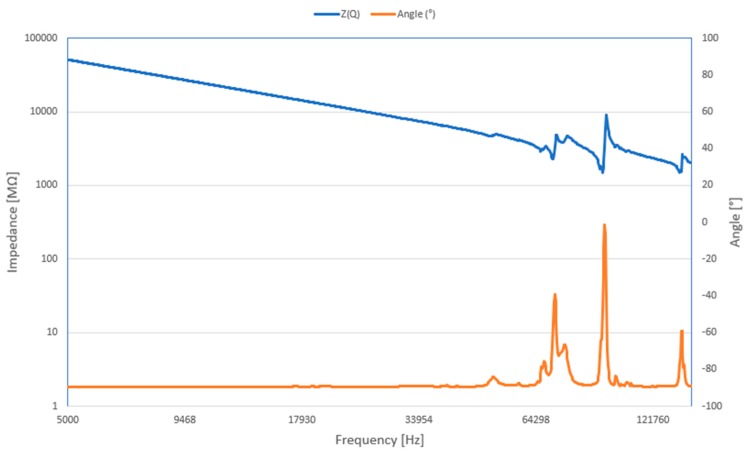
An impedance and phase angle measurement results of piezoelectric transducer, when signal driven electrode is connected.

Secondly, the electric impedance versus frequency was measured with an impedance analyzer 6500B (Wayne Kerr Electronics Ltd., Bognor Regis, UK). The setup is shown in Figure 12. One signal driven electrode was connected to the impedance analyzer. The results are presented in Figure 13. It can be seen that the measured resonant frequency is similar to the frequency obtained during the numerical simulation. The dominant measured resonance frequency value is at 92.1 kHz. Furthermore, the results are approximately the same as a result of numerical simulations (~91 kHz). The errors can be caused by various reasons such as the inaccuracy of the material properties and dimensions, soldered wires, and others.

**Figure 14:**
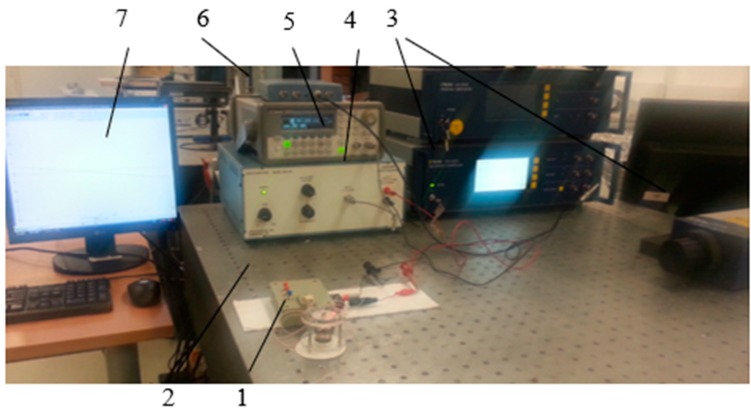
Experimental setup for measurement of resolution of the angular rotation: 1—USM with spherical rotor; 2—controller; 3—Polytec Laser Doppler Vibrometer system OFV512/5000; 4—linear amplifier EPA-104; 5—signal generator Agilent 33220A; 6—oscilloscope PicoScope 3424; 7—PC with a PicoScope and Polytec software.

**Figure 15:**
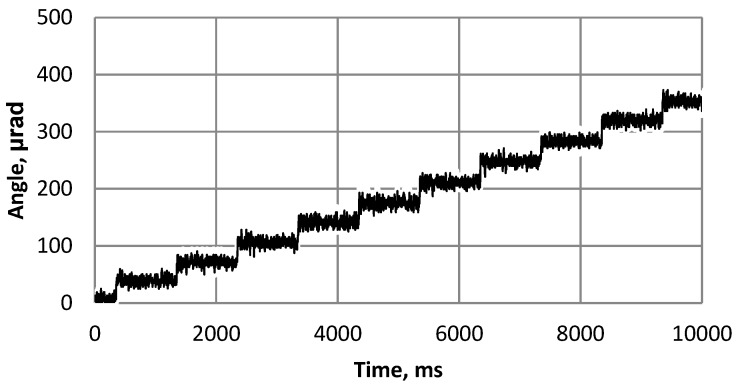
Measured resolution results of angular motion of the sphere.

Finally, a harmonic burst type signal of frequency 92 kHz and amplitude 60 V consisted of 20 cycles was applied and resolution of the angular step type rotation of the sphere was measured using laser 1D Polytec vibrometer OFV5000/512. The experimental setup can be seen in Figure 14. A laser beam was pointed to the rotor, which reflected the laser signal. The reflected signal was processed and calculations were done. The results of this measurement are shown in Figure 15. The measured average resolution of the step is 30 µrad.

## 5. Conclusions

The novel design concept of a 3-DOF piezoelectric USM of rotational motion was proposed and numerical simulations, as well as experimental studies, were performed. The results are summarized as follows:

1. Amplitudes of the contact points vibrations at the resonant frequency depend on the segment number of the top surface electrode and the highest length of the major axis is obtained when the top electrode was divided into six sections.
2. The direction of the sphere rotation can be controlled by switching the corresponding segment of the electrode.
3. A prototype of USM was fabricated and experiments were performed. Resonant frequencies and modal shapes calculated during numerical simulation demonstrate good agreement with the results obtained during the experimental measurement.
4. The sphere angular rotation step measurement was performed and the resolutaion of 30 µrad was achieved.

## Figures and Tables

**Table 1:** The properties of the material used for numerical simulation.

Material Property	Piezoceramics PZT-4
Young Modulus, *GPa*	63
Poisson coefficient	0.31
Density, kg/m^3^	7500
Dielectric permittivity, 10^3^ F/m	ε_11_ = 1.48, ε_22_ = 1.48, ε_33_ = 1.3
Piezoelectric coupling matrix, 10^−12^ C/N	d_31_ = −123, d_32_ = −123, d_33_ = 289, d_24_ = 496, d_15_ = 496
Compliance matrix, 10^−12^ m^2^/N	c_11_= 12.3, c_21_ = −4.05, c_31_ = −5.31, c_22_ = 12.3, c_32_ = −5.31, c_33_ = 15.5, c_44_ = 39, c_55_ = 39, c_66_ = 32.7

**Table 2:** Properties of the materials and dimensions of components used for simulation.

Components	Flange	Contact Element
Material properties	Aluminum 6061	Aluminum oxide (Al_2_O_3_).
Young’s modulus, GPa	68.9	215
Poison’s ratio	0.33	0.21
Density, kg/m^3^	2700	3950
Dimensions, mm	ODxIDxH = 30 × 14 × 1,5	1,5 × 2,5 × 1,5

**Table 3:** Dimensions of ellipses major and minor axes.

Components	3 Segments (µm)	6 Segments(µm)	9 Segments(µm)
Major axis *U_(xz_*_)_	24.24	27.31	11.66
Minor axis *U_(xz_*_)_	12.605	13.63	12.64
